# Influenza A(H5N8) Virus Similar to Strain in Korea Causing Highly Pathogenic Avian Influenza in Germany

**DOI:** 10.3201/eid2105.141897

**Published:** 2015-05

**Authors:** Timm Harder, Sebastian Maurer-Stroh, Anne Pohlmann, Elke Starick, Detlef Höreth-Böntgen, Karin Albrecht, Gunter Pannwitz, Jens Teifke, Vithiagaran Gunalan, Raphael T.C. Lee, Carola Sauter-Louis, Timo Homeier, Christoph Staubach, Carola Wolf, Günter Strebelow, Dirk Höper, Christian Grund, Franz J. Conraths, Thomas C. Mettenleiter, Martin Beer

**Affiliations:** Friedrich-Loeffler-Institut, Insel Riems, Germany (T. Harder, A. Pohlmann, E. Starick, D. Höreth-Böntgen, J. Teifke, C. Sauter-Louis, T. Homeier, C. Staubach, G. Strebelow, D. Höper, C. Grund, F.J. Conraths, T.C. Mettenleiter, M. Beer);; Nanyang Technological University, Singapore (S. Maurer-Stroh); Ministry of Health, Singapore (S. Maurer-Stroh);; Agency for Science, Technology and Research, Singapore (S. Maurer-Stroh, V. Gunalan, R.T.C. Lee);; County Veterinary Office, Anklam, Germany (K. Albrecht, G. Pannwitz);; Agency for Agriculture, Food Security and Fisheries of the German Federal State of Mecklenburg–Western Pomeranio, Rostock, Germany (C. Wolf)

**Keywords:** influenza A virus, H5N8 subtype, highly pathogenic, poultry, wild birds, zoonoses, viruses, influenza, Germany

## Abstract

Highly pathogenic avian influenza (H5N8) virus, like the recently described H5N8 strain from Korea, was detected in November 2014 in farmed turkeys and in a healthy common teal (*Anas crecca*) in northeastern Germany. Infected wild birds possibly introduced this virus.

Reassortant highly pathogenic avian influenza (HPAI) viruses of subtype H5N8 were introduced into South Korea in early 2014, possibly by virus-infected wild birds. The virus, which was spread widely by wild birds and within farming networks, caused major outbreaks of HPAI in poultry, was associated with deaths in aquatic wild birds, and spread to Japan (*1*–*4*). Related viruses were detected in China (*5*–*7*), but before early November 2014, the viruses had been confined to eastern Asia. This study sought to confirm the etiology of a major outbreak of HPAI (H5N8) on a turkey farm in northeastern Germany and to determine the virus’s possible origin.

## The Study

On November 3, 2014, a sudden increase in deaths among 16-week-old turkeys was noticed at an indoor turkey-fattening facility in northeastern Germany (*8*). The affected farm kept 31,000 turkeys and is situated in an area with low density of poultry ≈1.3 km east of Lake Galenbeck, a protected, internationally recognized nature reserve frequented by wild birds. The completely fenced farm is surrounded by fields and forest and has restricted access. The turkeys were kept in stables A and B, each with 3 units (A1–3 and B1–3), all connected by a corridor. On November 1 and 2, 2014, 0.5% of turkeys were found dead (expected number of deaths = 0) in unit A3, which is near the stable complex entrance. On November 3 and 4, the number of dead turkeys increased sharply (731 and 899, respectively; 18.4% and 22.6% daily mortality rates). When turkeys were culled on November 6, 2014, ≈300 turkeys were alive in A3 (93.4% cumulative mortality rate). In the adjacent unit, A2, onset of disease followed the course occurring in A3 with an increased number of deaths delayed by 1–2 days, but deaths never reached levels found in A3. Units A1, B1, and B2 had been little affected when all turkeys were culled (unit B3 was not in use).

RNA extracted from swab samples of viscous mucus in the oropharynx of dead turkeys and from an organ mixture was positive for influenza A(H5N8) virus by using reverse transcription quantitative PCR, conventional reverse transcription PCR, and sequencing (*9*). The polybasic hemagglutinin (HA) cleavage site sequence RNSPLRERRRKR*GLF indicated a highly pathogenic phenotype.

**Figure 1:**
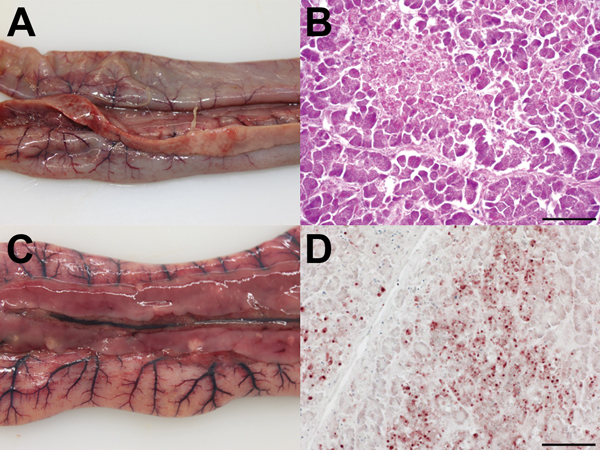
Pathomorphologic results for 2 dead turkeys infected with influenza A(H5N8) virus, Germany. A, C) Gross pathology showing acute, focally extensive to diffuse pancreatic necrosis with fibrinous serositis. B, D) Hematoxylin and eosin staining showing acute coagulative necrosis of the pancreas and multifocal staining within the exocrine pancreatic acini for influenza A virus nucleocapsid protein. Scale bars indicate 50 µm (B) and 100 µm (D).

Pathomorphologic examination of 2 turkeys revealed herds of pancreatic necrosis associated with fibrinous exudates, necrosis of ileocecal tonsils, and discrete petechiae in peri- and subepicardial locations. Heavily injected subserosal mesenteric vessels dominated the situs. Immunohistologic analysis confirmed systemic infection and revealed influenza virus nucleocapsid protein in 1) ganglions of the adrenal medulla, 2) ependymal cells of the central nervous system (associated with marked lymphocytic meningitis and perivascular cuffing), 3) thymus epithelia, and 4) epithelia of the exocrine pancreas (Figure 1).

**Figure 2:**
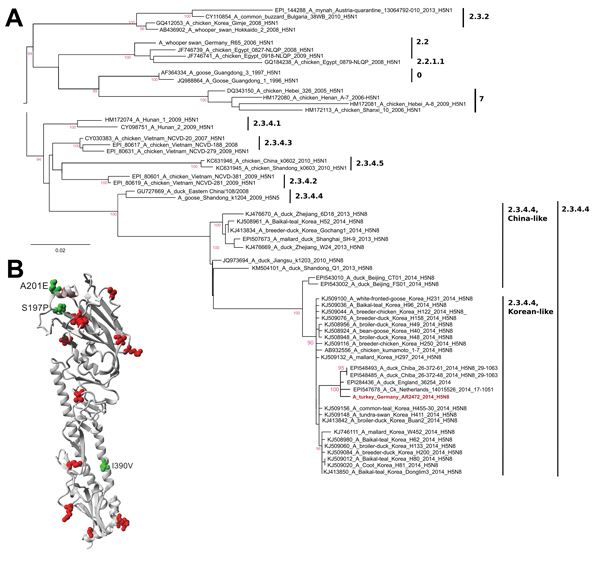
Phylogenetic analysis of hemagglutinin (HA) 1 nucleotide sequences of highly pathogenic avian influenza viruses subtype H5 from Southeast Asia and Germany. Insert shows the structural model of the HA protein of the German H5N8 isolate AR2472/14. A) Nucleotide sequences encoding the membrane-distal part of the HA1 of influenza A(H5N8) viruses were retrieved from public databases, aligned by using MAFFT (http://mafft.cbrc.jp/alignment/software) and phylogenetically analyzed by using a maximum-likelihood approach (best fit model: K3Pu+G4) implemented in IQ-Tree (http://www.cibiv.at/software/iqtree) (*12*). Numbers at nodes represent surrogates of branching robustness obtained by an ultrafast bootstrap approach (*12*). Scale bar indicates nucleotide substitutions per site. B) Model of an HA monomer of AR2472/14 with PDB:3FKU used as template. Green depicts unique mutations distinguishing this virus from other South Korea–origin avian influenza (H5N8) viruses; red indicates additional substitutions relative to the closest vaccine candidate within clade 2.3.4.4. (A/Sichuan/26221/2014 [H5N6]).

A virus isolate (A/turkey/Germany-MV/AR2472/2014; AR2472/14) was obtained in embryonated chicken eggs and in a chicken hepatocyte culture (LMH, ATCC CRL-2117). Full-genome sequencing (using Sanger technology) and nontargeted next-generation sequencing, followed by phylogenetic analyses of the sequences (EPI_ISL_167140), confirmed a close relationship of all 8 segments of AR2472/14 to HPAI virus subtype H5N8 of clade 2.3.4.4 from South Korea (Figure 2, panel A). Within this clade, 2 sister lineages (A/breeder duck/Korea/Gochang1/2014 and A/Baikal teal/Korea/Donglim3/2014 [Donglim3/14]) can be distinguished. The AR2472/14 isolate forms a cluster with the Donglim3/14 lineage (*10*), but the derived coding sequences of all segments of AR2472/14 show 14 unique amino acids differing from those of Donglim3/14 (Table).

**Table:** Unique amino acid changes found in A/turkey/Germany-MV/R2472/2014 influenza virus compared with A/Baikal teal/Korea/Donglim3/2014 influenza virus*

Segment no.	EpiFlu accession no.	Protein	Mutation
1	EPI548431	PB2	V338I
			R497S
			K699R
2	EPI548430	PB1	T57K
3	EPI548429	PA	T162I
			A343V
			R385K
4	EPI544756	HA	S197P
			A201E
			I390V
5	EPI548427	NP	D51N
6	EPI544759	NA	A190T
			M470T
7	EPI548426		–
8	EPI548428	NS1	V65L

*Numbering according to A/Baikal teal/Korea/Donglim3/2014; sequences were deposited in the GISAID sequence database (http://gisaid.org). PB, polymerase basic; PA, polymerase acidic; HA, hemagglutinin; NP, nucleoprotein; NA, neuraminidase; NS, nonstructural; –, no mutation.

Three unique amino acid substitutions were found in HA (S197P, A201E, and I390V, numbered according to influenza A virus subtype H5N8) and modeled in the HA structure (*11*) (Figure 2, panel B). Although S197P is near the receptor-binding site, no HA mutations involve residues previously associated with host specificity, and key residues are in the typical avian configuration: E(190)202, G(225)237, Q(226)238, G(228)240. A201E and S197P are close to antigenic site Sb and could have limited influence on the antigenic profile of AR2472/14. Substitution I390V is in the binding interface of some of the universally neutralizing HA stalk antibodies. Compared with the most geographically widespread H5 clade in birds in East Asia (2.3.2.1), several alterations occur in key antigenic regions, possibly promoting spread of the new clade variant 2.3.4.4. Genetically, A/Sichuan/26221/2014 (H5N6) appears to be the closest human vaccine candidate in preparation (*13*). Although this influenza A virus subtype differs from AR2472/14 by 19 HA mutations, the mutations are scattered over different regions of the structure (Figure 2, panel B), and putative cross-protection requires confirmation. 

In the neuraminidase protein, unique substitutions A190T and M470T were detected in AR2472/14, but on the basis of the deduced protein sequence, AR2472/14 is expected to be sensitive to current neuraminidase inhibitors. In the internal genes, which are shared with the Korean H5N8 subtype, matrix 2 (M2) N31 may confer amantadine resistance, and a C-terminal extension in nonstructural protein 1 masks the PDZ binding motif otherwise involved in host interactions. The sequences of the polymerase basic (PB) 1, nucleoprotein (NP), matrix 1 and 2, and nonstructural (NS) 1 proteins were similar to the prototype sequence of A Donglim3/14; however, the matrix protein had no changes, and the PB1, NP, and NS1 genes had 1 aa change each. Three unique changes were identified in each of the PB2 and polymerase acidic proteins (Table).

Applying restriction measures according to European Union directive 94/2005 (*14*), such as culling affected flocks, has been effective in stopping further spread of this virus to other poultry farms. Epidemiologic investigations revealed no definite route of introduction of the virus but have excluded incursion by infected turkey eggs or poults; contaminated water, feed, or litter; and vehicles or persons having contact with infected premises in South Korea or East Asia. Introduction by infected wild birds, perhaps facilitated by contaminated litter, feed, water, fomites, or other substance cannot be excluded because an internationally recognized site frequented by wild birds is near the affected turkey farm.

Shortly before the start of the outbreak, large numbers of migratory birds were observed on harvested fields near the premise. Fecal wild bird samples collected from the environment around the farm were negative for influenza A(H5N8) virus RNA. However, a swab specimen obtained from a healthy common teal (*Anas crecca*) shot from a flock of wild ducks on the island of Ruegen, Germany, on November 17, 2014, showed positive results for HPAI (H5N8) virus.

Since this study began, HPAI H5N8 subtype outbreaks in poultry and infections in wild birds have been reported in Europe (the Netherlands, England, Italy, Hungary, Sweden and Germany), Asia (Russia and Japan), and North America (Canada and the United States). On the basis of available sequences, strains from Japan differ only slightly from influenza A(H5N8) viruses from Europe, suggesting that the common ancestor of this new H5N8 subtype variant likely emerged in Asia before recently spreading to Europe (Figure 2, panel A).

## Conclusions

The HPAI outbreak in northeastern Germany in November 2014 resulted from an HPAI (H5N8) subtype virus, represented by isolate AR2472/14, which is closely related to H5N8 subtype viruses that have hitherto been confined to the Far East. Fourteen unique coding mutations of AR2472/14 show differences between this virus and previous isolates from South Korea, but the mutations are shared with the recent H5N8 isolate A/duck/Chiba/26-372-61/2014 from Japan. Epidemiologic and phylogenetic data collected so far are insufficient to establish definite pathways of introduction into Germany. All possible routes, including relay transmission by subclinically infected wild birds, must be thoroughly examined. Enhanced active monitoring of sites frequented by aquatic wild birds and waterfowl is also recommended.

Technical Appendix Sequences from the GISAID EpiFluDatabase (http://www.gisaid.org) on which this research is based.
